# Effectiveness of targeted social and behavior change communication on maternal health knowledge, attitudes, and institutional childbirth: a cluster-randomized trial in Jimma Zone, Ethiopia

**DOI:** 10.1093/eurpub/ckae220

**Published:** 2025-01-06

**Authors:** Lakew Abebe Gebretsadik, Abebe Mamo, Zewdie Birhanu Koricha, Sudhakar Morankar

**Affiliations:** Department of Health, Behavior, and Society, Faculty of Public Health, Institute of Health, Jimma University, Jimma, Ethiopia; Department of Health, Behavior, and Society, Faculty of Public Health, Institute of Health, Jimma University, Jimma, Ethiopia; Department of Health, Behavior, and Society, Faculty of Public Health, Institute of Health, Jimma University, Jimma, Ethiopia; Department of Health, Behavior, and Society, Faculty of Public Health, Institute of Health, Jimma University, Jimma, Ethiopia

## Abstract

Maternal mortality remains a critical global health challenge, with 95% of deaths occurring in low-income countries. While progress was made from 2000 to 2015, regions such as Ethiopia continue to experience high maternal mortality rates, impeding the achievement of the sustainable development goal to reduce maternal deaths to 70 per 100 000 live births by 2030. This study evaluated the effectiveness of a Social and Behavior Change Communication (SBCC) intervention to improve maternal health behaviors. A community-randomized trial was conducted in three districts of Jimma Zone, rural Ethiopia, involving 5057 women. Sixteen primary healthcare units were randomly assigned to either the intervention (SBCC) or control (standard care) group. Data on socio-demographics, antenatal care (ANC) visits, maternal health knowledge, attitudes, and institutional childbirth rates were collected at baseline and endline. Statistical analyses included *t*-tests, effect sizes, and generalized estimating equations. The intervention group demonstrated significant improvements. Maternal health knowledge increased from 5.68 to 7.70 (*P* < .001, effect size = 0.34), attitudes improved from 37.49 to 39.73 (*P* < .001, effect size = 0.29), and ANC visits rose from 3.27 to 4.21 (*P* < .001, effect size = 0.50). Institutional childbirth rates increased from 0.52 to 0.71 (*P* < .001, effect size = 0.18). ANC attendance (B = 0.082, *P* = .002) and positive attitudes (B = 0.055, *P* < .001) were significant predictors of institutional childbirth. The SBCC intervention significantly enhanced maternal health knowledge, attitudes, ANC utilization, and institutional childbirth rates, highlighting the value of community-based strategies in improving maternal health behaviors.

## Introduction

Maternal mortality represents a critical global health crisis, with low-income countries accounting for 95% of maternal deaths [1–3]. Despite notable progress from 2000 to 2015, recent reports indicate stagnation or declines in maternal health indicators in some regions [4]. In Ethiopia, high maternal mortality rates impede progress toward the sustainable development goals (SDGs), particularly the target of reducing maternal deaths to 70 per 100 000 live births by 2030 [2, 5–7].

Initiatives such as the Health Extension Program and the Women’s Development Army (WDA) have been implemented to address these challenges; however, rural populations continue to face significant barriers to accessing maternal health services [8–12].

Innovative strategies are crucial for overcoming these obstacles. Maternal waiting homes provide essential housing near healthcare facilities, helping to mitigate geographic barriers. Additionally, Social and Behavior Change Communication (SBCC) initiatives promote institutional childbirth and improve maternal health outcomes [13–15]. SBCC programs aim to transform community attitudes through targeted messaging and local engagement, particularly important in rural areas with limited healthcare access [16, 17].

This study evaluates a targeted SBCC intervention in rural Ethiopia, which involves collaboration among religious leaders, WDA members, and health extension workers. The intervention includes training sessions, health education sessions, community dialogues, home visits, and supervision, all aimed at improving childbirth rates and fostering positive attitudes toward maternal health. By addressing knowledge gaps and promoting healthy norms, this approach seeks to provide valuable insights and scalable solutions for enhancing maternal health behaviors and health in resource-limited settings.

## Methods

### Study registration

The trial was registered on 3 October 2017, at ClinicalTrials.gov (registration number NCT03299491) [13].

### Study setting

The study was carried out in the Jimma Zone, situated 356 km southwest of Addis Ababa, Ethiopia, which has a rural population of 3.4 million. Three districts—Gomma, Seka-Chekorsa, and Kersa—were chosen due to their relatively high population density, challenges in maternal health, availability of primary healthcare units (PHCUs), and the active involvement of local actors and authorities. The populations of these districts range from 216 151 to 279 639. Maternal healthcare services in these areas are provided through a network of PHCUs, each consisting of a central health center and five satellite health posts that serve an average of 5000 people per health post. The PHCUs are supported by health extension workers (HEWs), who play a vital role in connecting informal and formal healthcare systems. They are crucial for enhancing access to prenatal and postnatal care, thus improving maternal health in rural areas.

### Study design

This study employed a pragmatic, two-arm, cluster-randomized trial design to evaluate the impact of a targeted SBCC intervention on the uptake of institutional childbirth services. Sixteen PHCUs were randomly assigned to two groups: Arm 1 (8 PHCUs) received the social and behavioral change communication (SBCC) intervention and Arm 2 (8 PHCUs) acted as the control group and received standard care.

The sample size calculation aimed to detect a 15% difference in institutional childbirth rates between the intervention and control arms, while accounting for the effects of clustering. Using a formula specific to cluster-randomized trials, we ensured that the study would have adequate power and precision:


$$n=\frac{\left(Z1-\alpha /2+Z1-\beta {)}^{2}\;*\;(P1\left(1-P1\right)+P2\left(1-P2\right)\right)}{\Delta P^{2}}.$$

For α = 0.05, Z1−α/2=1.96; for β = 0.20, Z1−β = 0.84.

Plug in the values:


$$n=\frac{\left(1.96+0.84{)}^{2}\;*\;(0.4(1-0.4)+0.55(1-0.55)\right)}{(0.15{)}^{2}}$$

n=169.64

Adjust for clustering:


$$\mathrm{Ncluster}=\left(1+\left(m-1\right)\;*\;\mathrm{ICC}\right)\;*\;n$$

$$\mathrm{Ncluster}=\left(1+\left(8-1\right)\;*\;0.1\right)\;*\;169.64=288.39$$

Nperarm=m * Ncluster=8 * 288.39

Nperarm=2307.12

The total sample size for both arms: Ntotal=2 * Nperarm=2 * 2307.12 = 4614.24.

To account for a potential 10% non-response rate, the final sample size was increased to 5,120 participants to ensure statistical robustness.

### Study participants and sampling procedures

**Figure 1.**
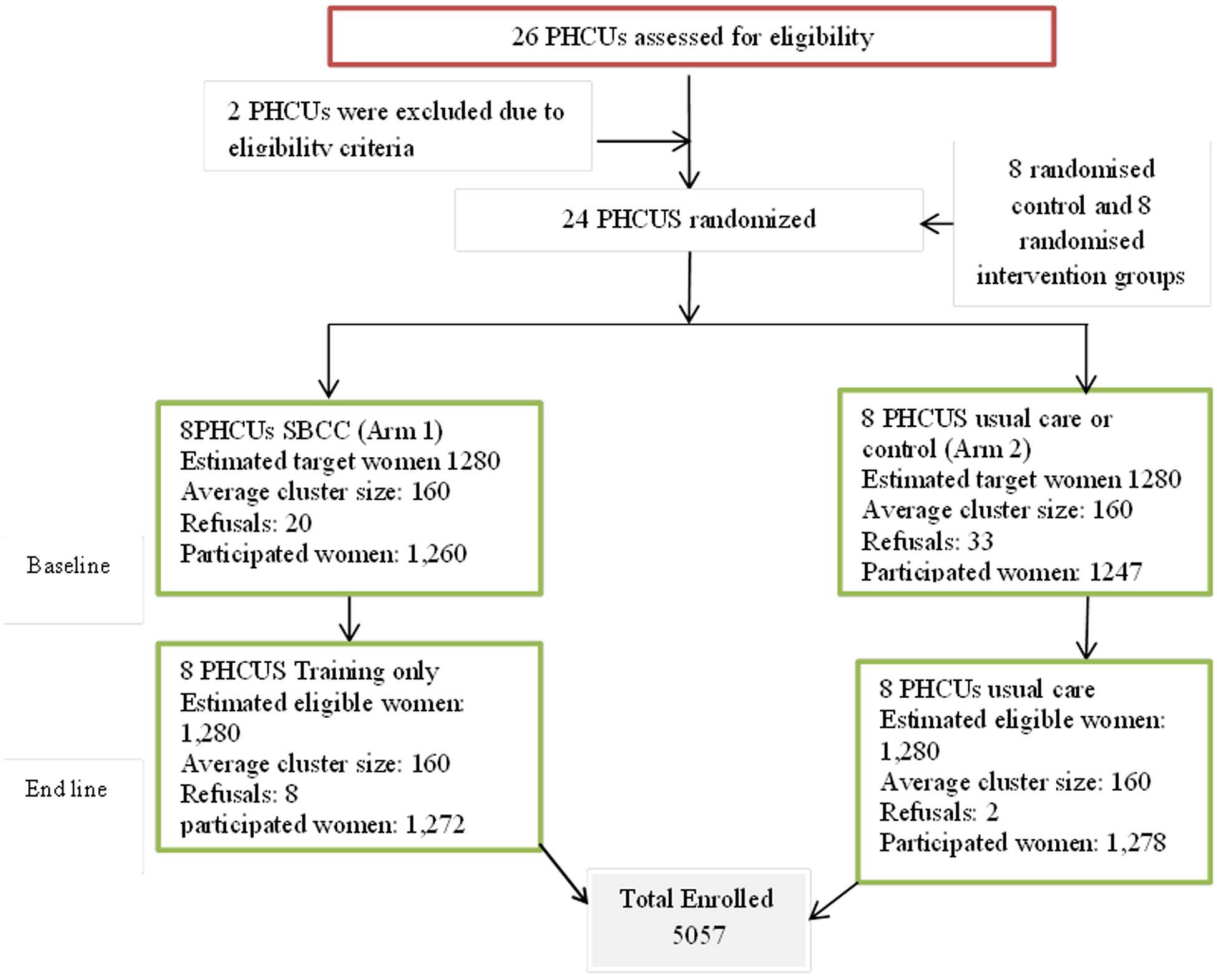
A flowchart illustrating trial profile, participant recruitment, randomization, and outcome assessments of SBCC intervention.

The sampling process used pre-existing census lists of women who had given birth within the previous year. Using STATA software, participants were randomly selected to achieve a representative sample of 5057 women, encompassing both baseline and endline assessments, with an average of 160 women per cluster per arm. The participant selection process and flow are illustrated in (Fig. 1).

### Randomization and blinding

Clusters were randomly assigned to intervention or control groups, with individual participants acting as observation units. Using STATA's random number generator, we allocated the 16 clusters to each arm. Data collectors and evaluators were blinded to group assignments to mitigate potential bias, thereby reducing the risk of performance bias and contamination [14].

### Intervention process

The intervention was effectively implemented across three districts in the Jimma Zone, impacting approximately 511 869 people, including 113 277 women of reproductive age (15–49 years). Ninety-one HEWs, 7363 WDAs, and 143 religious leaders were trained, achieving an 87% training completion rate.

### Key components included

Training of community health actors (CHAs): HEWs and WDAs received specialized training using authoritative manuals from WHO, UNICEF, and USAID to promote maternal health.

Community outreach and education: CHAs conducted proactive door-to-door outreach to emphasize the importance of institutional childbirth and prenatal care, effectively addressing cultural misconceptions.

Ongoing support: Monthly training sessions and updates from HEWs ensured strict adherence to the intervention and promptly addressed challenges.

Health education sessions: Regular sessions conducted by HEWs engaged the community and provided essential support to pregnant women.

### Usual care

The control arm received standard care, which included existing community-government collaborations focused on improving healthcare access and delivering regular health education sessions aimed at identifying and supporting pregnant women. This included monthly follow-ups to monitor community needs and reinforce maternal health messaging [15].

### Data collection and quality assurance

The questionnaire was translated into Afan Oromo and Amharic, with back-translation to ensure accuracy. It was adapted from the JHPIEGO toolkit and the 2016 Ethiopia Demographic and Health Survey. Data collection utilized Samsung tablets with Open Data Kit (ODK) software, conducted by 20 trained data collectors. A pre-test in the Mana district ensured streamlined procedures and data quality.

### Variables and measurements

The primary dependent variable was the occurrence of institutional childbirth, coded as 1 for delivery at a healthcare facility and 0 for non-institutional deliveries. Independent variables included antenatal care (ANC) utilization, maternal attitudes, knowledge about maternal health, and socioeconomic and demographic characteristics. ANC utilization was measured by the total number of ANC visits completed. Maternal attitudes were assessed using a 15-item Likert-type scale (scored from 15 to 45), and knowledge was measured with 10 binary questions (scored as knowledgeable [1] or not knowledgeable [0]), resulting in composite scores from 0 to 10. Socioeconomic and demographic variables included occupation, education, marital status, family income, and travel time to healthcare facilities. Age and family size were measured as continuous variables.

### Data analysis

Descriptive statistics were used to summarize socioeconomic and demographic characteristics. Frequencies and percentages were reported for categorical variables, while means and standard deviations were calculated for continuous variables. To evaluate within-group changes over time, paired *t*-tests were applied, and independent *t*-tests were used to compare mean differences between the intervention and control groups. Generalized estimating equations (GEE) were employed to analyze the effect of the SBCC intervention on institutional childbirth rates while adjusting for longitudinal data clustering and accounting for potential confounders. Analysis outputs, including coefficients and odds ratios, demonstrated that ANC attendance and maternal attitudes significantly influenced the likelihood of institutional childbirth.

## Results

### Sociodemographic characteristics of study participants at baseline and endline

The sociodemographic characteristics of the study participants indicate that most were aged 20–29, with an average age of 27. Notably, 58.5% of those in the baseline control group had no formal education while the endline intervention group saw a significant increase in participants with a high school education or higher, reaching 10.6%.

Most participants identified as housewives, though this group experienced a slight decline by the endline, particularly in the control group (73.2%). Conversely, the endline intervention group reported a slight increase in other occupations (22.9%). Marital status remained stable, with most individuals being married, ranging from 96.5% to 98.2%. Across all groups, family size was generally large, with a mean size ranging from 5.5 to 5.8 members. Families with five or fewer members were roughly equal to those with more than five, although the baseline control group showed a slight tendency toward larger families (52.0%). Income distribution exhibited shifts, particularly in the third quartile, where both endline groups experienced a significant increase (70.9% in the control group and 70.0% in the intervention group).

Transportation methods to delivery locations varied. Most control group participants (70.7%) traveled on foot. By the end of the study, there was an increased reliance on public transport and ambulances, particularly in the intervention group, where ambulance usage rose to 23.0%. These changes in transportation highlight the improved accessibility and utilization of healthcare facilities, possibly due to the intervention’s emphasis on emergency preparedness and transport planning. For further details, refer to Table 1.

**Table 1.** Sociodemographic characteristics of study participants at baseline and endline

Characteristics	Measurement	Baseline control (*N* = 1247)	Baseline intervention (*N* = 1260)	Endline control (*N* = 1278)	Endline intervention (*N* = 1272)
Age category	M (SD)	27.77 (5.84)	27.50 (5.66)	27.22 (5.72)	27.28 (5.37)
10–19	*N* (%)	69 (5.5)	82 (6.5)	89 (7.0)	67 (5.3)
20–29	*N* (%)	711 (57.0)	744 (59.0)	770 (60.3)	796 (62.6)
30–39	*N* (%)	419 (33.6)	402 (31.9)	383 (30.0)	376 (29.6)
40–49	*N* (%)	48 (3.8)	32 (2.5)	36 (2.8)	33 (2.6)
Education level					
No formal education	*N* (%)	730 (58.5)	648 (51.4)	663 (51.9)	570 (44.8)
Primary school	*N* (%)	474 (38.0)	523 (41.5)	526 (41.2)	567 (44.6)
High school+ above	*N* (%)	43 (3.4)	89 (7.1)	89 (7.0)	135 (10.6)
Women’s occupation					
Housewife	*N* (%)	979 (78.5)	968 (76.8)	933 (73.2)	971 (77.1)
Others	*N* (%)	268 (21.5)	292 (23.2)	341 (26.8)	289 (22.9)
Marital status					
Not living together	*N* (%)	22 (1.8)	36 (2.9)	43 (3.4)	44 (3.5)
Married	*N* (%)	1225 (98.2)	1224 (97.1)	1235 (96.6)	1228 (96.5)
Family size					
≤5	*N* (%)	598 (48.0)	674 (53.5)	633 (49.5)	674 (53.0)
>5	*N* (%)	649 (52.0)	586 (46.5)	645 (50.5)	598 (47.0)
Mean (SD)	M (SD)	5.83 (2.1)	5.59 (2.07)	5.75 (2.08)	5.6 (2.01)
Income level quartiles					
First quartile	*N* (%)	562 (45.1)	558 (44.3)	95 (7.4)	97 (7.6)
Second quartile	*N* (%)	501 (40.2)	462 (36.7)	208 (16.3)	226 (17.8)
Third quartile	*N* (%)	126 (10.1)	155 (12.3)	906 (70.9)	891 (70.0)
Fourth quartile	*N* (%)	58 (4.7)	85 (6.7)	69 (5.4)	58 (4.6)
Means of transportation to delivery place					
By foot	*N* (%)	882 (70.7)	731 (58.0)	843 (66.0)	641 (50.4)
Stretcher, animal	*N* (%)	70 (5.6)	72 (5.7)	93 (7.3)	59 (4.6)
Public transport	*N* (%)	111 (8.9)	175 (13.9)	161 (12.6)	280 (22.0)
Ambulance	*N* (%)	184 (14.8)	282 (22.4)	181 (14.2)	292 (23.0)

### Impact of SBCC intervention

The proportion of childbirths at health institutions showed a slight increase in the control group, with the mean rising from 0.51 (SD = 0.50) at baseline to 0.53 (SD = 0.50) at endline. In contrast, the intervention group experienced a more substantial increase, with the mean rising from 0.52 (SD = 0.50) at baseline to 0.71 (SD = 0.46) at the endline. The improvement in the intervention group indicates that the SBCC strategy effectively promoted institutional delivery and has the potential for broader use in similar contexts. This change was statistically significant, with a mean difference of 0.18 (pooled SD = 0.48), a *t*-value of −9.509 (df = 2530), *P* < .001, and an effect size of −0.18.

ANC visits showed a minor decrease in the control group, with the mean slightly dropping from 3.26 (SD = 0.86) at baseline to 3.23 (SD = 1.02) at endline. The intervention group demonstrated a notable increase in ANC visits, with the mean rising from 3.27 (SD = 0.87) at baseline to 4.21 (SD = 0.97) at endline. The observed increase in ANC visits within the intervention group may reflect enhanced awareness and motivation driven by targeted SBCC messages that underscored the importance of continuous maternal healthcare. This improvement was highly significant, with a mean difference of 0.94 (pooled SD = 0.98), a *t*-value of −24.862 (df = 2530), *P* < .001, and an effect size of −0.48.

Knowledge scores remained stable in the control group, increasing from a mean of 5.68 (SD = 3.10) at baseline to 5.92 (SD = 2.19) at endline. Conversely, the intervention group experienced a significant increase in knowledge, with the mean rising from 5.68 (SD = 2.82) at baseline to 7.70 (SD = 1.82) at the endline. The marked increase in knowledge in the intervention group suggests that the SBCC intervention successfully enhanced participants’ understanding of maternal health and institutional childbirth. This resulted in a mean difference of 1.78 (pooled SD = 2.00), a *t*-value of −22.325 (df = 2530), *P* < .001, and an effect size of −0.44.

Attitude scores showed a modest improvement in the control group, increasing from a mean of 37.19 (SD = 4.10) at baseline to 37.86 (SD = 3.34) at endline. In contrast, the intervention group experienced a more substantial rise, with the mean increasing from 37.49 (SD = 3.91) at baseline to 39.73 (SD = 3.26) at the endline. The increase in positive attitudes within the intervention group indicates a favorable shift toward institutional childbirth, likely due to the SBCC’s influence on participants' perceptions and motivation. This change was statistically significant, with a mean difference of 2.24 (pooled SD = 3.60), a *t*-value of −14.307 (df = 2530), *P* < .001, and an effect size of −0.28. For more information, see Table 2.

**Table 2.** Impact of the intervention on knowledge, attitudes, ANC visits, and institutional childbirth

Variable	Study phase	Study group	*N*	Mean	SD	*t*-test	*P*-value	Effect size
Childbirth at health institution	Baseline	Control	1247	0.51	0.500	−0.501	.62	−0.01
		Intervention	1260	0.52	0.500			
	Endline	Control	1278	0.53	0.499	−9.509	<.001	−0.18
		Intervention	1272	0.71	0.456			
ANC	Baseline	Control	1247	3.26	0.86	−0.289	.77	−0.01
		Intervention	1260	3.27	0.87			
	Endline	Control	1278	3.23	1.02	−24.862	<.001	−0.48
		Intervention	1272	4.21	0.97			
Knowledge	Baseline	Control	1247	5.68	3.10	0.000	1.00	0.00
		Intervention	1260	5.68	2.82			
	Endline	Control	1278	5.92	2.19	−22.325	<.001	−0.44
		Intervention	1272	7.70	1.82			
Attitude	Baseline	Control	1247	37.19	4.10	−1.874	.06	−0.07
		Intervention	1260	37.49	3.91			
	Endline	Control	1278	37.86	3.34	−14.307	<.001	−0.28
		Intervention	1272	39.73	3.26			

### Determinants of institutional childbirth: insights from GEE analysis

The GEE analysis examined various factors influencing the likelihood of institutional childbirth. The intercept for the threshold (childbirth = 0) was significant (B = 2.522, *P* < .001), indicating a substantially higher probability of institutional childbirth [Exp(B) = 12.459, 95% confidence interval (CI) 7.444–20.854].

Each additional antenatal care visit was positively associated with institutional childbirth (B = 0.082, *P* = .002), increasing the odds by 8.5% (Exp(B) = 1.085, 95% CI 1.029–1.144). This association underscores the pivotal role of consistent ANC visits in influencing mothers’ decisions to deliver at healthcare facilities. Positive attitudes toward institutional childbirth significantly influenced the outcome (B = 0.055, *P* < .001), resulting in a 5.7% increase in the odds (Exp(B) = 1.057, 95% CI 1.038–1.076).

However, knowledge regarding institutional childbirth did not significantly affect the outcome (B = 0.039, *P* = .221, Exp(B) = 1.039, 95% CI 0.977–1.106). Additionally, the interaction between knowledge and attitude was not statistically significant (B = 0.001, *P* = .516, Exp(B) = 1.001, 95% CI 0.999–1.003). These findings suggest that while knowledge is important, positive attitudes and consistent ANC visits may play more direct roles in encouraging institutional childbirth. These results underscore the critical role of antenatal care visits and positive attitudes in promoting institutional childbirth (see Table 3).

**Table 3.** Generalized estimating equation analysis of the intervention’s effect on institutional childbirth

Parameter	B	Std. Error	Sig	Exp(B)	95% Wald Confidence Interval for Exp(B)	
					Lower	Upper
Threshold (childbirth = 0)	2.522	0.2628	0.000	12.459	7.444	20.854
Antenatal care	0.082	0.0268	0.002	1.085	1.029	1.144
Knowledge	0.039	0.0317	0.221	1.039	0.977	1.106
Attitude	0.055	0.0092	0.000	1.057	1.038	1.076
Knowledge × attitude	0.001	0.0011	0.516	1.001	0.999	1.003
(Scale)	1.000					

Dependent variable: Childbirth at home = 0; childbirth at health institution = 1.

Model: Threshold, antenatal care, knowledge, attitude, knowledge * attitude.

## Discussion

This study provides compelling evidence of the effectiveness of community-based interventions in enhancing maternal healthcare outcomes. Our findings align with recent findings in similar settings, highlighting the importance of culturally tailored educational approaches that resonate with the community’s values and health needs [16, 17]. Prior studies have demonstrated the pivotal role of community health workers (CHWs) in developing countries, leading to significant improvements in ANC attendance and institutional delivery rates by fostering a connection between healthcare services and local traditions [18, 19].

In our intervention group, participants exhibited a substantial increase in ANC visits and institutional deliveries compared to the control group. This success is particularly noteworthy in rural Ethiopia, where analogous strategies have yielded positive results in countries such as India and Tanzania. Our approach, integrating WDAs and HEWs, fosters community trust and promotes skilled birth attendance. Encouraging health-seeking behaviors among rural populations is vital for enhancing maternal and child health outcomes and aligns with efforts to bridge gaps between healthcare systems and rural communities [20–22].

Leveraging local community resources can significantly improve maternal healthcare utilization and outcomes, as demonstrated in Kenya [23]. Our study observed considerable gains in maternal health knowledge and attitudes among participants, aligning with recent studies emphasizing the effectiveness of community-based educational initiatives in advancing maternal health in Ethiopia through targeted behavioral interventions that address local knowledge gaps and cultural perceptions [19, 24, 25]. Through maternal health-specific SBCC interventions, we cultivated positive attitudes toward healthcare practices, fostering timely health-seeking behaviors critical for advancing maternal and child health in these communities. This aligns with literature suggesting the efficacy of community-driven health education programs across diverse sociocultural contexts and emphasizes the need for continued community engagement to achieve sustainable improvements [26–28].

The impact of SBCC strategies on ANC attendance and childbirth outcomes is particularly significant. The higher rates of institutional deliveries and ANC attendance among intervention participants support international initiatives focused on reducing maternal mortality through community-centered, culturally appropriate interventions [29–31]. Expanding access to trained birth attendants and promoting safe delivery practices are vital elements of global initiatives and highlight the importance of involving trusted community figures to build rapport and encourage institutional delivery.

While our study highlights improvements in maternal healthcare utilization and outcomes, it also underscores the necessity of addressing systemic barriers and practical challenges. Sustained investment in healthcare infrastructure, workforce training, and oversight is essential to meet the increased demand generated by effective SBCC programs [32, 33]. Reliable supply chains and well-equipped facilities are critical for maintaining and building upon the gains achieved through community-based interventions. Additionally, overcoming these practical barriers will guarantee that communities maintain access to high-quality healthcare, even after interventions end [34].

Additionally, our findings emphasize the importance of community engagement and the integration of local cultural practices. Building trust and achieving successful health interventions require a harmonious blend of traditional practices with modern healthcare services and can help overcome resistance to institutional healthcare among rural populations [35, 36]. The involvement of respected community figures, such as WDAs and HEWs, contributed to improved maternal health outcomes, reinforcing the growing body of evidence supporting the effectiveness of SBCC strategies in low-income settings like rural Ethiopia [37, 38].

Integrating insights from diverse studies and fostering partnerships with local communities and healthcare providers offer promising pathways to advance maternal health goals. Sustaining these efforts requires significant investment in health systems and policies to improve maternal and child health globally and enable adaptable, scalable interventions for other regions [39, 40]. Continued investment in infrastructure, workforce capacity, and sustainable funding is crucial for advancing holistic approaches to maternal healthcare. With proper support, SBCC interventions can reduce maternal and neonatal mortality, contributing to global health equity and maternal health goals aligned with the SDGs.

### Strengths of the study

This study showcases its strengths through a robust methodology and impactful findings. The cluster-randomized trial design enhances external validity and eliminates selection bias by assigning 16 PHCUs. This led to a significant 19% increase in institutional childbirth rates, marking a crucial advancement in reducing maternal mortality and demonstrating that similar interventions can succeed in low-resource settings.

The study’s effectiveness is boosted by an 87% completion rate in training CHWs and the use of digital tools for reliable data collection. Future research will tackle social desirability bias in self-reported data and offer recommendations to address systemic barriers to maternal health.

### Limitations of the study

The study has notable limitations. Social desirability and performance bias may have impacted participants' and providers' behaviors due to their awareness of group allocations. Data collection was limited to baseline and endline measurements, lacking intermediate data points.

The results primarily apply to rural districts in Ethiopia and similar developing regions, as contextual factors may vary. Although improvements in attitudes and knowledge were observed, knowledge alone did not predict positive institutional childbirth outcomes, highlighting the need to explore practical factors that promote behavior change.

## Conclusion

This study highlights the effectiveness of community-based SBCC interventions in enhancing maternal health outcomes in rural Ethiopia. The interventions led to increased ANC visits and higher institutional childbirth rates, showcasing the vital roles of CHWs, WDAs, and HEWs.

Despite these gains, challenges such as inadequate healthcare infrastructure, supply chain issues, and the need for better workforce training remain. The findings indicate that simply providing knowledge is not enough to drive behavior change; addressing barriers like access to care and cultural alignment is crucial.

The results suggest that culturally tailored SBCC interventions can be scaled in low-resource settings. Continued investment in healthcare infrastructure and workforce development, along with strong partnerships and local leadership engagement, will **f**urther improve maternal health outcomes globally.

## Data Availability

The datasets analyzed during this study are available upon request from the corresponding author.
